# Postoperative pain management for circumcision; Comparison of frequently used methods

**DOI:** 10.12669/pjms.36.2.505

**Published:** 2020

**Authors:** Caglar Munevveroglu, Mehmet Gunduz

**Affiliations:** 1 Caglar Munevveroglu, Department of Pediatric Surgery, Istanbul Medipol University, Medical Faculty, Istanbul, Turkey; 2 Mehmet Gunduz, Department of Pediatrics, Istanbul Medipol University, Medical Faculty, Istanbul, Turkey

**Keywords:** Analgesia methods, CHEOPS, Circumcision, Postoperative pain

## Abstract

**Objective:**

To determine the ideal method for postoperative pain management after circumcision by comparing the most frequently used different methods like; dorsal penile block, caudal epidural block, subcutaneous ring block, intravenous paracetamol and intravenous tramadol HCl.

**Methods:**

Between May 1^st^ 2015 to May 1^st^ 2016, 500 children between 2-10 year old were circumcised at the department of pediatric surgery of Istanbul Medipol University Health Care Practice & Research Center Sefakoy Hospital. Five groups were formed according to postoperative analgesia methods which were planned to be compared; Group-I. penile block, Group-II. Caudal epidural block, Group-III. subcutaneous ring block, Group-IV as intravenous paracetamol and Group-V as intravenous tramadol HCl. In order to evaluate the postoperative pain levels of children, Children’s Hospital Eastern Ontario Pain Scale (CHEOPS) was filled at 30, 60, 120, 180 minutes after circumcision by a researcher who does not know which method was applied.

**Results:**

No significant difference is found between the groups (p>0.05). In the statistical analysis, no significant difference was found in the effect of analgesia methods on CHEOPS scores between 30, 60, 120 and 180 minutes (p>0.05). In parallel with this result, no significant difference was found in the effect of heart beat rates and respiration rate averages between 30, 60, 120 and 180 minutes (p>0.05).

**Conclusion:**

It has been shown that none of the five method has any superiority in reducing pain after circumcision and that all five methods can be used. However, we think that side effects of regional anesthesia and systemic analgesic applications should not be ignored.

## INTRODUCTION

Circumcision is the oldest and most common surgical procedure in the world and managing such a common surgery basically requires patient safety, reliability, rapid recovery and adequate pain management.^1^ Numerous studies have assessed the postoperative pain, analgesia and circumcision relationship until today.^2^ But publications addressing the relationship between circumcision and pain often deal with pain relief during surgery and do not consider the postoperative effect of the methods they use. During and after surgery inadequate pain relief may have long-term complications like altered sensory processing and painful stimuli response.^1^,^3^,^4^

In our daily medical practice, we frequently encounter circumcision demands for boys who are both newborn and older. One of the biggest concerns of the parents in this respect is the state of pain which is the main factor for determining the comfort of children after circumcision. Based on our clinical experience, we observe that the families of children who are suffering pain after being circumcised have panic and do not know what to do. However, unfortunately no standard pain relief method has been established to reduce pain after circumcision. Systemic NSAID’s, opioid analgesics or local anesthetic techniques are being used for postoperative pain management. On the other hand systemic analgesic agents like NSAID’s and opioid analgesics can cause gastrointestinal (ie; dyspepsia, bleeding), genitourinary (ie; acute kidney failure and tubulary necrosis), dermatological (ie; erythema multiforme) and pulmonary (asthma provacation) adverse side effects and other invasive techniques also can cause local (ie; haematoma), systemic and neurological (ie; motor block, delayed micturition) adverse side effects.^5^-^7^ So, none of the above are innocent methods so determining the best one will be very helpful for pain management after circumcision.

In this study, we aimed to determine the ideal method for postoperative pain management after circumcision by comparing the most frequently used methods like; dorsal penile block, caudal epidural block, subcutaneous ring block, intravenous paracetamol and intravenous tramadol HCl.

## METHODS

After obtaining the hospital ethical committee approval (Dated: November 7, 2018) we collected patients’ data retrospectively. Five groups were formed according to postoperative analgesia methods which were planned to be compared; Group-I. penile block (following negative aspiration, to the sites of 10 and 2 o’clock at the penis base, with dose of 0.2 ml/kg 0.25% bupivacaine), Group-II. Caudal epidural block (with 0.2 ml / kg 0.25% bupivacaine to be entered from the sacral hiatus using the Tuohy needle in the left side decubitus position), Group-III. Subcutaneous ring block (with 0.2 ml/kg 0.25% bupivacaine to be injected subcutaneously at the penis base circumferentially), Group-IV as intravenous paracetamol (15 mg / kg during the operation) and Group-V. intravenous tramadol HCl (1 mg / kg during the operation). After the approval of the parents of the children are taken, between 01.05.2015 and 01.05.2016 one pediatric surgeon planned to perform circumcision operation at the department of pediatric surgery of Istanbul Medipol University Health Care Practice & Research Center Sefakoy Hospital under general anesthesia with the guillotine method, 500 children between 2-10-year-old were randomly assigned to the groups in equal numbers. Patients with any additional health problems identified during the history and physical examination are excluded from the study. Without premedication, after patients are taken to the operation room, IV cannulation is performed and anesthesia induction is made with Propofol 2-3 mg/kg and fentanyl 2µg/kg. After performing appropriate laryngeal mask for the patient, sevoflurane, oxygen and nitrogen is used for the maintenance of anesthesia. Children’s ages, weights and duration of operation were recorded. During or after the circumcision procedure, the appropriate planned analgesia method to the child’s group is applied (caudal epidural block, IV paracetamol and IV tramadol HCl were administered by anesthesiologist, dorsal penile block and subcutaneous ring block were administered by pediatric surgeon) and in order to evaluate the postoperative pain levels of children, Children’s Hospital Eastern Ontario Pain Scale (CHEOPS) (Table-I) was filled at 30, 60, 120, 180 minutes after circumcision by a researcher who does not know which method was applied. At the same time patients’ heart beat rates and minute respiratory rates were recorded. The data obtained from the results were evaluated and compared statistically. Age, weight and operation time were compared with one-way variance analysis.

**Table-I:** Children’s Hospital Eastern Ontario Pain Scale (CHEOPS).

		Score
Cry	No cry	1
	Moaning or crying	2
	Scream	3
Facial	Smiling	1
	Composed	2
	Grimace	3
Verbal	Positive	1
	None/Other complaints	2
	Pain complaints	3
Torso	Neutral	1
	Shifting/Tense/Shivering	2
Touch	Not touching wound	1
	Reach/Touch/Grab wound	2
Legs	Neutral	1
	Squirm/Kicking	2
	Standing/Crouching/Kneeling	3

Other data analysis was performed using IBM SPSS Version 18.0 statistical package program. The significance level is determined as 0.05 in all analyzes. Since the homogenity (Levene test, p>0.05) is considered among the studied groups, the assumption of the normality of distribution (Kolmogorov-Smirnov, p>0.05) is fulfilled and because of there are five groups at the study, the ANOVA test was used to analyze the data. As a result of this analysis, post-hoc test statistics were applied to determine the source of significant difference between groups.

## RESULTS

The mean age, weight and duration of operation of the cases are given in Table-II. When these variables were evaluated, no significant difference is found between the groups (p>0.05). In the statistical analysis, no significant difference was found in the effect of analgesia methods on CHEOPS scores between 30, 60, 120 and 180 minutes (p>0.05). In parallel with this result, no significant difference was found in the effect of heart beat rates and respiration rate averages between 30, 60, 120 and 180 minutes (p>0.05) (Table-III). None of the children included in the study had any surgical complications like infection, bleeding or secondary phimosis. None of the Group-I patients had any adverse side effects like haematoma which is the most frequent side effect of penile block. None of the Group-II patients had any adverse side effects such as haematoma, irritability, motor block (limb weakness) or delayed micturition and none of the patients from all groups had any drug reaction or any systemic adverse side effect which requires readmission.

**Table-II:** Demographic data of the participants according to the groups.*

	Group-I	Group-II	Group-III	Group-IV	Group-V
Age (year)	4.8 ± 2	4.5 ± 2.1	4.6 ± 2.5	5.2 ± 2.5	4.9 ± 2.4
Weight (kg)	17.8 ± 9	18.5 ± 9.5	18 ± 9.8	17.9 ± 8.9	19.2 ± 9.1
Duration of operation (min)	13 ± 1.2	13 ± 1.3	12 ± 1.2	14 ± 1	14 ± 1.1

* Mean value ± Standard deviation.

**Table-III:** CHEOPS, HBR and RR averages according to the measurement time of the groups.*

	Group-I	Group-II	Group-III	Group-IV	Group-V
CHEOPS 30´	9.05±2.4	8.96±2.74	9.25±3.04	9.15±2.88	8.83±2.46
HBR 30´	109.1±17.06	104.2±16.02	115.35±15.7	103.95±14.72	106.85±15.99
RR 30´	29.1± 3.95	28.4±3.42	32.8±2.56	27.94±2.85	28.8±3.26
CHEOPS 60´	7.85±2.1	7.56±2.01	7.9±2.59	7.95±2.58	7.45±2.41
HBR 60´	106.85±14.8	102.4±11.5	107.5±10.14	101.85±13.08	104.9±16.7
RR 60´	29±2.65	28.1±2.54	29.08±2.48	27.98±3.02	28.7±2.85
CHEOPS 120´	6.55±1.95	6.45±1.23	6.85±1.69	6.65±1.84	6.42±1.07
HBR 120´	103.65±11.54	103.03±11.21	106.35±12.93	100.05±13.7	103.04±15.08
RR 120´	27.05±3.35	26.98±3.42	28.83±3.67	25.1±3.1	26.88±3.56
CHEOPS 180´	6.6±2.3	6.28±1.56	6.65±1.95	6.05±1.69	5.95±0.81
HBR 180´	106.2±11.7	103.5±13.64	108.15±12.49	102.2±12.52	104.5±16.94
RR 180´	27.65±3.57	26.93±3.48	28.12±3.06	25.97±2.86	27.16±3.21

* Mean Value ± Standard deviation.

## DISCUSSION

Circumcision is one of the most common surgical procedures of pediatric surgeons. Contrary to the past, a good pain control is aimed during and after circumcision nowadays to increase the comfort of the child and to reduce the psychological effects of this period. If postoperative analgesia can be successfully achieved, it may prevent the adverse effects of pain. In this way, patient anxiety, morbidity, length of hospitalization and cost are reduced.^8^ To provide analgesia after circumcision, caudal epidural block, dorsal penile block, subcutaneous ring block, iv, oral rectal paracetamol are among the frequently used methods. Different assessment methods such as the Neonatal Infant Pain Scale (NIBPS) and CHEOPS are used for evaluation of pain in children.^9^ CHEOPS method is used in our study.

In daily pediatric medical practice, intravenous paracetamol administration is a frequently used method to provide analgesia, whether postoperatively or not. Rare side effects associated with the use of paracetamol includes anaphylaxis, liver disorders, hypotension, and tachycardia. Similarly, intravenous tramadol HCl is an analgesic tool with a practical use to reduce pain in postoperative periods. Side effects include sweating, nausea, anaphylaxis and liver disorders. In the light of recent developments in the field of anesthesia, regional anesthesia techniques are used safely and effectively in combination with general anesthesia in children.^9^ Caudal epidural block, dorsal penile block and subcutaneous ring block are frequently used for pain management of circumcision. Aside from the mechanical side effects such as the occurrence of hematoma, there are also potential side effects due to the chemical structure of the local anesthetics. These may include complications such as methemoglobinemia and also convulsion and cardiac arrest due to the use of local anesthetics which include adrenaline. There is no reported significant complication of ring-shaped superficial infiltration anesthesia (subcutaneous ring block).^10^,^11^ In our study, we compared the efficacy of intravenous tramadol HCl and intravenous paracetamol in addition to regional anesthesia methods and we did not encounter any complication due to anesthesia applications.

In many previous studies, the efficacy of various methods for postoperative analgesia after circumcision were compared with each other and different results were obtained. Unfortunately, there is no common point accepted by everyone. For example; Vater et al.^12^ found that the caudal epidural block was more advantageous after their study, while Weksler et al.^13^ did not find any difference between the two methods. In another study, White et al.^14^ suggested that dorsal penile block was more advantageous but on the other hand Tutuncu et al. suggested that dorsal penile block has less advantages than other methods which they compared.^6^

Fundamentally all circumcision methods can be grouped under four main headings. These are; dorsal slit, Sheldon method (Circumcision shield, Mogen Clamp etc.), special circumcision clamps and open surgical methods (dorsal slit + excision, Sleeve method, guillotine prepicial excision). In studies conducted to investigate the effects of analgesic agents after circumcision, it has been reported that surgical method also has an effect on postoperative analgesia. Therefore, in our study the same surgical method (guillotine method) was used in all cases. In the second step of our study, we aim to evaluate different anesthesia methods with different surgical procedures.

As pediatric surgeons, we are frequently faced with the desire for circumcision in our country due to religious and social traditions. It is the physicians’ duty to take the necessary measures to help the child overcome this process with as little as possible physical and psychological damage. In this way, families can spend this stressful period more easily and comfortably. For the post-circumcision period, the main concern of the families is the pain of their children. Unfortunately, circumcision, a procedure that is so frequently performed, does not yet have a definitive analgesia procedure for postoperative pain, and many different methods are used in many clinics. Our study was designed to eliminate this complexity and compared the most commonly used methods to reduce postoperative pain after circumcision. In the literature search, we have not found any previous studies comparing these five methods with each other.

## CONCLUSION

This study has shown that none of the five method has any superiority in reducing pain after circumcision and that all 5 methods can be used with peace of mind. However, we think that side effects of regional anesthesia and systemic analgesic applications should not be ignored.

## References

[1] Wang J, Zhao S, Luo L, Liu Y, Zhu Z, Li E (2018). Dorsal penile nerve block versus eutectic mixture of local anesthetics cream for pain relief in infants during circumcision:A meta analysis. PLoS One.

[2] Schröder A, Campbell FA, Farhat WA, Pippi Salle JL, Bägli DJ, Lorenzo AJ (2018). Postoperative pain and analgesia administration in children after urological outpatient procedures. J Pediatr Urol.

[3] Peters JW, Schouw R, Anand KJ, van Dijk M, Duivenvoorden HJ, Tibboel D Does neonatal surgery lead to increased pain sensitivity in later childhood?Pain. 2005;.

[4] Taddio A, Katz J (2005). The effects of early pain experience in neonates on pain responses in infancy and childhood. Paediatr Drugs.

[5] McGowan PR, May H, Molnar Z, Cunliffe M (1998). A comparison of three methods of analgesia in children having day case circumcision. Paediatr Anaesth.

[6] Tutuncu AC, Kendigelen P, Ashyyeralyeva G, Altintas F, Emre S, Ozcan R (2018). Pudendal nerve block versus penile nerve block in children undergoing circumcision. Urol J.

[7] Dalens B, Vanneuville G, Dechelotte P (1989). Penile block via the subpubic space in 100 children. Anesth Analg.

[8] Czarnecki ML, Hainsworth K, Simpson PM, Arca MJ, Uhing MR, Varadarajan J (2014). Is there an alternative to continuous opioid infusion for neonatal pain control?A preliminary report of parent/nurse-controlled analgesia in the neonatal intensive care unit. Paediatr Anaesth.

[9] Linder SL, Winner P (2001). Pediatric Headache. Med Clin North Am.

[10] American Academy of Pediatrics. Circumcision policy statement .Task Force on Circumcision (1999). Pediatrics.

[11] Cesur M, Alici HA (2005). Convulsion, cardiac arrest and death after penile blockade at home (case report). Anestezi Dergisi.

[12] Vater M, Wandless J (1985). Caudal or dorsal nerve block?Comparison of two local anesthetic techniques for postoperative analgesia following day-case circumcision. Acta Anesthesiol Scand.

[13] Weksler N, Attas I, Klein M, Rosenztsveig V, Ovaida L, Gurman GM (2005). Is penil block better than caudal epidural block for postcircumcision analgesia?. J Anesth.

[14] White J, Hasmson B, Richmond P, Proctor A, Curran J (1983). Postoperative analgesia for circumcision in children. Br Med J (Clin Res Ed).

